# Assessing joint line obliquity in valgus-producing high tibial osteotomy: A scoping review of the literature

**DOI:** 10.1016/j.jor.2025.01.021

**Published:** 2025-01-15

**Authors:** Tianshun Xie, Reinoud W. Brouwer, Inge van den Akker-Scheek, Hugo C. van der Veen

**Affiliations:** a Department of Orthopaedic Surgery, University of Groningen, University Medical Center Groningen, Groningen, the Netherlands; b Department of Orthopaedic Surgery, Martini Hospital, Groningen, the Netherlands

**Keywords:** Joint line obliquity, Measurement method, Radiographic technique, Measurement reliability, High tibial osteotomy

## Abstract

**Background:**

The variance in knee joint line obliquity (KJLO) measurement methods and radiographic techniques may contribute to the controversy on clinical consequences of KJLO after high tibial osteotomy (HTO).

**Purpose:**

To summarize currently used KJLO measurement methods, including their measurement reliability, and the radiographic techniques used in valgus-producing HTO.

**Methods:**

The databases PubMed, Embase, and Web of Science were searched from inception up to May 2023, to identify articles that measured KJLO on radiographs in valgus-producing HTO.

**Results:**

Thirty clinical articles were included. There were five different KJLO measurement methods reported, including joint line orientation angle by femoral condyles (JLOAF), joint line orientation angle by middle knee joint space (JLOAM), joint line orientation angle by tibial plateau (JLOAT), Mikulicz joint line angle (MJLA), and medial proximal tibial angle (MPTA), of which the JLOAT was the most commonly used. KJLO was measured on anteroposterior full-length standing radiographs with either single-leg or double-leg patient stance position, with no standardized bipedal distance on double-leg stance radiographs. Moderate-to-excellent measurement reliability was reported for intraobserver and interobserver MPTA, and good-to-excellent for intraobserver JLOAT and JLOAM and for interobserver JLOAT, JLOAM, and MJLA.

**Conclusion:**

There is no consensus on how to measure KJLO or on which radiographic technique should be used. When measuring joint line orientation angles on anteroposterior full-length double-leg stance radiographs, controlling the bipedal distance with feet together is suggested when possible. Future research is needed to determine the measurement differences between the five KJLO measurement methods and to identify the preferred, ideal one.

## 1 Introduction

Valgus-producing high tibial osteotomy is a powerful surgical procedure performed for medial knee osteoarthritis in patients with varus malalignment, aiming to realign the lower limb weight-bearing line from the affected medial knee compartment to the relatively healthy lateral side, slow down knee osteoarthritis progression, and postpone knee arthroplasty.^1^^,^^2^ However, this surgical process could introduce an increased knee joint line obliquity (KJLO) in the coronal plane.^3^, ^4^, ^5^

Excessive KJLO can lead to a notable rise in shear stress and a redistribution of contact stress within the knee joint.^6^, ^7^, ^8^ However, controversial evidence exists regarding the relationship between postoperative KJLO and patient-reported outcomes, status of medial knee cartilage, and long-term surgical survivorship subsequent to valgus-producing high tibial osteotomy.^9^ The variance in KJLO measurement methods and radiographic techniques used may contribute to this controversy, so evaluation of KJLO and hence decision-making remain difficult in patients with suspected excessive KJLO.

The purpose of this scoping review was to summarize currently used KJLO measurement methods, including their measurement reliability when possible, and the radiographic techniques used in valgus-producing high tibial osteotomy.

## 2 Methods

This review followed the Preferred Reporting Items for Systematic Reviews and Meta-analyses (PRISMA) guideline for scoping reviews.^10^

### 2.1 Search strategy

A literature search was conducted on February 18, 2023 in three online electronic databases: PubMed, Web of Science, and Embase, with an updated search on May 1, 2023. Articles were retrieved from the date of online database inception up to the search date. The search strategies of the three databases, which were optimized by a librarian, are shown in Table 1.

**Table 1:** Search strategy.

Online Database	Search String
PubMed	(“Osteoarthritis, Knee” [Mesh] OR “Knee” [Mesh] OR “Knee Joint” [Mesh] OR knee∗ [tiab]) AND (“Osteotomy” [Mesh] OR osteotom∗ [tiab]) AND (joint line obliquit∗ [tiab] OR joint line orientat∗ [tiab] OR jlo [tiab])
Web of Science	TS = “knee∗” AND TS = “osteotom∗” AND TS= (“joint line obliquit∗” OR “joint line orientat∗” OR “jlo”)
Embase	(“knee osteoarthritis”/exp OR “knee”/exp OR knee∗:ab,ti,kw) AND (“osteotomy”/exp OR osteotom∗:ab,ti,kw) AND (“joint line obliquit∗”:ab,ti,kw OR “joint line orientat∗”:ab,ti,kw OR jlo:ab,ti,kw)

### 2.2 Article selection and data extraction

Articles meeting the following criteria were included: KJLO was measured in patients planned for valgus-producing high tibial osteotomy, and the KJLO measurement method was clearly described. Articles on nonpatient research such as cadaveric studies and finite element analysis studies were excluded. No language restriction was used in the article selection.

Article selection and data extraction process: (1) duplicate articles were manually excluded from the search outcome; (2) title, abstract, and full text were independently assessed by two reviewers (TX and HV) by checking the predefined criteria; (3) relevant references of the included articles were manually searched for additional articles; (4) information on publication year, study location, separate patient groups, osteotomy techniques, stance position in filming, KJLO measurement method used and its measurement reliability when possible, and preoperative KJLO mean values were extracted from each included article (TX); (5) two reviewers (TX and HV) achieved consensus on included articles and extracted information in discussion meetings, and a third reviewer (IvdAS) was consulted if there was disagreement between the two reviewers.

### 2.3 Grading measurement reliability

The measurement reliability of radiological parameters is usually evaluated by the intraclass correlation coefficient (ICC) between two measurements of the same observer (intraobserver reliability) and between the measurements of two observers (interobserver reliability). ICC values < 0.5, 0.5–0.75, 0.75–0.9, and >0.9 represent the grades of poor, moderate, good, and excellent measurement reliability, respectively.^11^

## 3 Results

**Fig. 1:**
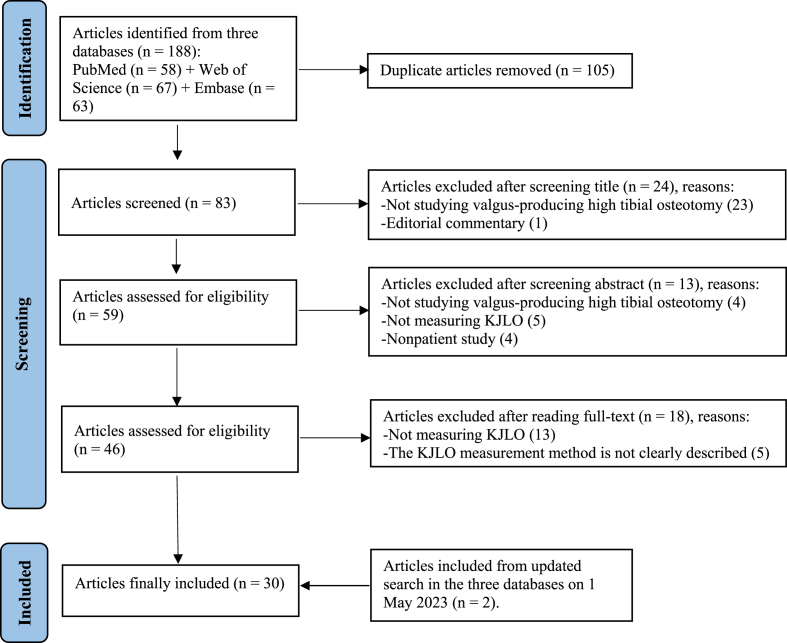
Literature Selection Process by PRISMA flowchart.

The search strategy identified 188 articles, containing 105 duplicates. Of the remaining 83 articles, 55 were excluded based on the predefined inclusion and exclusion criteria. The updated searching and screening resulted in 2 additional articles. In the end, 30 clinical articles were included. A flowchart of the article selection process is presented in Fig. 1. The extracted information from the included articles is summarized in Table 2 and below.

**Table 2:** Summary of included articles.

Authors (year, location)	Osteotomy techniques (separate patient groups)	KJLO measurement methods	Radiographic techniques	KJLO mean values, Preoperative *(postoperative)*	KJLO measurement reliability	
					Intraobserver ICC	Interobserver ICC
Babis et al. (2008, USA)^4^	LCW HTO	JLOAT	Double-leg stance	−0.5° (*NM*)	NM	NM
Victor et al. (2014, Belgium)^22^	HTO	JLOAT	Double-leg stance with feet together	NM (*NM*)	NM	NM
Lee KM et al. (2015, Korea)^33^	MOW HTO	JLOAM	NM	0.3° (*4.4°)*	Good	Good
Oh et al. (2016, Korea)^17^	MOW HTO	JLOAT	Double-leg stance with patella distance equal to shoulder width	−0.7° *(1.3°)*	Excellent	Excellent
Kim CW et al. (2017, Korea)^14^	MOW HTO	JLOAM	Double-leg stance	−0.4° *(3.2°)*	NM	Good
Akamatsu et al. (2018, Japan)^28^	MOW HTO (postoperative MPTA>95°/≤95°)	JLOAF	Single-leg stance	0.7°/−0.1° *(5.7°/3.2°)*	NM	NM
Goshima et al. (2019, Japan)^13^	MOW HTO	JLOAT	Double-leg stance with patella distance equal to shoulder width	−2.3° *(1.4°)*	Excellent	Excellent
Park JY et al. (2019, Korea)^37^	MOW HTO	JLOAM	NM	−0.8° *(2.9°)*	Good	Good
Bartholomeeusen et al. (2020, Belgium)^12^	MOW HTO	JLOAT	Double-leg stance with medial sides touching upper legs, knees, and ankles	1.81° *(3.46°)*	NM	NM
Goto et al. (2020, Japan)^31^	LCW HTO	MPTA	NM	NM *(96.6°)*	NM	NM
Kim JE et al. (2020, Korea)^32^	MOW HTO	JLOAT	NM	0.79° *(2.72°)*	Good	Excellent
Kubota et al. (2020, Japan)^29^	MOW HTO	JLOAT	Single-leg stance	1.1° *(2.6°)*	Excellent	Good
Park JG et al. (2020, Korea)^36^	MOW HTO	JLOAT	NM	0.5° *(3.6°)*	Excellent	Good
Song et al. (2020, Korea)^38^	MOW HTO	JLOAT	NM	NM (*NM*)	Excellent	Excellent
Kim GW et al. (2021, Korea)^15^	MOW HTO	JLOAF	Double-leg stance	NM (5.5°/0.9°)	NM	NM
Lee SJ et al. (2021, Korea)^34^	MOW HTO	JLOAT	NM	2.1° *(3.3°)*	Good-to-excellent	Good-to-excellent
Miyazaki et al. (2021, Japan)^35^	MOW HTO	JLOAT	NM	−1.0° *(2.4°)*	Good-to-excellent	Good-to-excellent
Park JG et al. (2021, Korea)^19^	MOW HTO (preoperative MPTA ≥85°/<85°)	JLOAT	Double-leg stance	2.2°/−0.4° *(5.3°/3.5°)*	Excellent	Excellent
Park JG et al. (2021, Korea)^18^	MOW HTO	JLOAT	Double-leg stance	0.7° *(4.5°)*	Excellent	Excellent
Akamatsu et al. (2022, Japan)^3^	MOW HTO	JLOAF	Single-leg stance	1.4° *(6.3°)*	NM	NM
Hiramatsu et al. (2022,Japan)^23^	MOW HTO	JLOAT	Double-leg stance with knees at shoulder width	−0.66° *(3.0°)*	NM	NM
Kubota et al. (2022, Japan)^30^	MOW HTO (rod/MPTA)	JLOAT	Single-leg stance	1.3°/−0.7° *(3.4°/1.4°)*	Good-to-excellent	Good-to-excellent
Kawashima et al. (2022, Japan)^24^	MOW HTO	JLOAT	Double-leg stance	−0.5° *(2.8°)*	Good-to-excellent	Good-to-excellent
Kim JS et al. (2022, Korea)^16^	MOW HTO (postoperative MPTA 85°–90°/90°–93°/93°–95°/95°–102°)	JLOAT	Double-leg stance	−1.02°/−1.01°/−0.66°/0.06° *(-0.10°/0.26°/1.57°/5.14°)*	Excellent	Good-to-excellent
		MPTA		83.81°/84.75°/84.46°/84.63° *(89.12°/92.06°/93.52°/96.04°)*	Moderate-to-excellent	Moderate-to-good
Park SB et al. (2022, Korea)^25^	MOW HTO (unilateral/primarily bilateral/secondarily bilateral treated limbs)	JLOAT	Double-leg stance	1.2°/1.7°/1.1° *(3.1°/3.3°/2.7°)*	Excellent	Excellent
Rosso et al. (2022, Italy)^5^	MOW HTO	MJLA	Double-leg stance	88.3° *(90.6°)*	NM	Excellent
		MPTA		85.1° *(91.5°)*		Excellent
Sohn et al. (2022, Korea)^20^	MOW HTO (postoperative MPTA ≥95°/<95°)	JLOAT	Double-leg stance with feet together	3.5°/0.7° *(6.0°/3.7°)*	Excellent	Good
Tseng et al. (2022, Taiwan)^21^	MOW HTO	JLOAT	Double-leg stance	−0.7° (*NM*)	Good	Good
Abs et al. (2023, France)^27^	MOW HTO	JLOAT	Double-leg stance	3.0° *(5.6°)*	Good	Good
Jeong et al. (2023, Korea)^26^	MOW HTO	JLOAT	Double-leg stance	NM (*NM*)	Good	Good

a Knee joint line obliquity (KJLO); Medial opening wedge high tibial osteotomy (MOW HTO); Lateral closing wedge high tibial osteotomy (LCW HTO); Medial proximal tibial angle (MPTA); Mikulicz joint line angle (MJLA); Not mentioned (NM).

b Intraclass correlation coefficient (ICC) is graded following Koo et al.’s guideline.^11^

c All studies use anteroposterior full length standing radiographs.

d For joint line orientation angle by femoral condyles (JLOAF), joint line orientation angle by middle knee joint space (JLOAM) and joint line orientation angle by tibial plateau (JLOAT), a positive value (+) indicates a medial opening angle, a negative value (−) indicates a lateral opening angle.

### 3.1 KJLO radiographic techniques

All studies used an anteroposterior full-length standing radiograph for KJLO measurement. Variation was seen in the standing position for single-leg stance or double-leg stance at filming and in bipedal distance on double-leg stance radiographs.

Eighteen clinical studies (18/30, 60.0 %) used the double-leg stance position at filming,^4^^,^^5^^,^^12^, ^13^, ^14^, ^15^, ^16^, ^17^, ^18^, ^19^, ^20^, ^21^, ^22^, ^23^, ^24^, ^25^, ^26^, ^27^ three with bipedal distance: Sohn et al.^20^ and Victor et al.^22^ controlled bipedal distance with both feet together, Bartholomeeusen et al.^12^ defined hip joint adduction until the touching of medial sides of the upper legs, knees, and ankles. Four clinical studies used single-leg stance position at filming (4/30, 13.3 %).^3^^,^^28^, ^29^, ^30^ The remaining eight clinical studies did not mention any stance position details (8/30, 26.7 %).^31^, ^32^, ^33^, ^34^, ^35^, ^36^, ^37^, ^38^

### 3.2 KJLO measurement methods

**Fig. 2:**
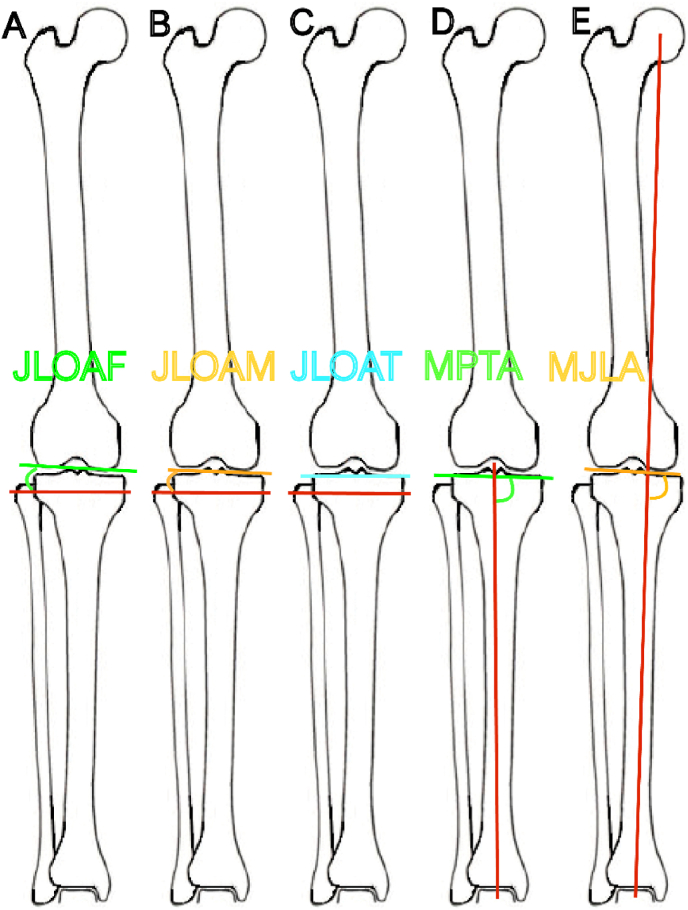
Illustration of KJLO Measurement Methods **A.** Joint line orientation angle by femoral condyles (JLOAF) is formed by the knee joint orientation line measured by the tangential line of the femoral condyles (green) and the ground line (red)^28^. **B.** Joint line orientation angle by middle knee joint space (JLOAM) is formed by the knee joint orientation line measured by the line connecting the midpoints of medial and lateral knee joint space (yellow) and the ground line (red)^33^. **C.** Joint line orientation angle by tibial plateau (JLOAT) is formed by the knee joint orientation line measured by the tangential line of the tibial plateau (blue) and the ground line (red)^22^. **D.** Medial proximal tibial angle (MPTA) is the medial angle between the tangential line of the tibial plateau (green) and the tibial mechanical axis (red)^16^. **E.** Mikulicz joint line angle (MJLA) is the medial angle between the middle knee joint space line (yellow) and the lower limb weight-bearing line (red).^5^ The weight-bearing line connects the femoral head centre and ankle joint centre.^52^

Five different KJLO measurement methods were reported, including joint line orientation angle by femoral condyles (JLOAF), joint line orientation angle by middle knee joint space (JLOAM), joint line orientation angle by tibial plateau (JLOAT), Mikulicz joint line angle (MJLA), and medial proximal tibial angle (MPTA). For clarification purposes, these five KJLO measurement methods are illustrated in Fig. 2. Twenty-one clinical studies used JLOAT to measure KJLO (21/30, 70.0 %),^4^^,^^12^^,^^13^^,^^17^, ^18^, ^19^, ^20^, ^21^, ^22^, ^23^, ^24^, ^25^, ^26^, ^27^^,^^29^^,^^30^^,^^32^^,^^34^, ^35^, ^36^^,^^38^ three used JLOAM (3/30, 10.0 %),^14^^,^^33^^,^^37^ three used JLOAF (3/30, 10.0 %),^3^^,^^15^^,^^28^ and one used MPTA (1/30, 3.3 %).^31^ Two clinical studies performed two KJLO measurement methods to assess KJLO (2/30, 6.7 %), one using JLOAT and MPTA,^16^ and the other using MJLA and MPTA.^5^

### 3.3 KJLO measurement reliability

Twenty-two clinical studies reported ICC outcomes of the KJLO measurement method used.^5^^,^^13^^,^^14^^,^^16^, ^17^, ^18^, ^19^, ^20^, ^21^^,^^24^, ^25^, ^26^, ^27^^,^^29^^,^^30^^,^^32^, ^33^, ^34^, ^35^, ^36^, ^37^, ^38^ Moderate-to-excellent measurement reliability was found for intraobserver and interobserver MPTA, and good-to-excellent for intraobserver JLOAT and JLOAM and for inter-observer JLOAT, JLOAM, and MJLA. There is a lack of reporting on intraobserver measurement reliability in measuring MJLA. No intraobserver or interobserver measurement reliability was reported in measuring JLOAF.

## 4 Discussion

The most important finding of this review is that the literature shows large variability in KJLO measurement methods and radiographic techniques used, which implies there is no consensus on which measurement method or radiographic technique should be used to assess KJLO.

Although JLOAT is the most commonly used KJLO measurement method, it is reported to be influenced by single-leg and double-leg stance positions as well as by bipedal distance in the double-leg stance position. According to Paley et al.,^39^ for healthy individuals, JLOAT measures 0° at the single-leg stance position and at the double-leg stance position with feet together. It reaches 3° lateral inclination at the double-leg stance position with a bipedal distance equal to pelvis width.^39^ Lee et al.^40^ found that a 10-cm bipedal distance increase could introduce a 3.7° JLOAT mean measurement change on anteroposterior full-length double-leg stance radiographs. Rosso et al.^5^ indicated that JLOAT was an unreliable KJLO measurement method, as measurement could be affected by the leg position relative to the ground. Since the three joint line orientation angles (JLOAT, JLOAM, JLOAF) are all formed by the ground line, it is reasonable to speculate that bipedal distance may also influence measurements of JLOAM and JLOAF. Hence to use joint line orientation angles for measuring KJLO on double-leg stance radiographs, a key procedure is to control and standardize the bipedal distance. The present review recommends using the at-attention stance position with feet together when physiologically possible. In this way, the measurements of joint line orientation angles on double-leg stance radiographs could be compared to their measurements on single-leg stance radiographs.

Whether single-leg and double-leg stance positions and bipedal distance in the double-leg stance position influence MJLA measurement remains unclear. Although studies have reported that the MPTA measurement was not affected by stance position, whether this measurement is influenced by bipedal distance on double-leg stance radiographs has not been identified. Bardot et al.^41^ and Yazdanpanah et al.^42^ found no statistically significant differences in MPTA measurements between single-leg and double-leg stance positions on anteroposterior full-length standing radiographs (p > 0.05). This finding could be explained by MPTA being measured based on the anatomical geometry of the tibial bone, thus the measurement is independent of the patient's stance position at filming. Unlike the measurements of joint line orientation angles, the measurements of MJLA and MPTA do not take the ground line into account. A reasonable hypothesis is that bipedal distance does not influence measurements of MJLA and MPTA. Future research is needed to verify this hypothesis.

There is no consensus on the preferred, ideal radiographic technique to be used for measuring KJLO in anteroposterior full-length single-leg stance or double-leg stance radiographs. In the included studies of this review, double-leg stance radiographs are used more frequently than single-leg stance radiographs, though each has its respective advantages and deficiencies. Na et al.^43^ and Hiranaka et al.^44^ reported that single-leg stance radiographs may be a superior radiographic technique for assessing dynamic lower limb alignment, as they are better at illustrating the loaded knee condition during gait by only providing weight-bearing on the affected knee joint. Conversely, Specogna et al.^45^ found that single-leg stance radiographs did not provide more representative measurements describing the condition of knee joint under dynamic load, and recommended using double-leg stance radiographs in surgical assessment for medial knee osteoarthritis. Double-leg stance radiographs provide a comparison of radiographic features between the affected knee and its contralateral side, and patients with severe pain and/or instability of the affected knee joint may be unable to take a single-leg stance radiograph.

As the included studies lack ICC outcome reporting on intraobserver and interobserver for the JLOAF and intraobserver for the MJLA, it is not yet possible to identify a superior method from the five KJLO measurement methods based on their measurement reliability.

Factors affecting knee joint space width may influence measurement of JLOAM and MJLA. According to the definitions, JLOAM and MJLA are formed by the knee joint orientation line that measures the middle knee joint space, so confounding factors affecting knee joint space width may need to be taken into consideration in measuring them, such as meniscus and cartilage thickness, knee osteoarthritis severity grade, lateral knee laxity, and medial knee tightness.^46^, ^47^, ^48^ Research is needed to find out how these confounding factors influence KJLO measurements.

In the included clinical studies, the heterogeneity of the radiographic techniques used makes it difficult to give a comprehensive comparison of the preoperative KJLO mean values between the five KJLO measurement methods. As mentioned, single-leg stance position and double-leg stance position, including bipedal distance, could influence measurements of JLOAT, JLOAM, and JLOAF. In addition, some included studies only provide the preoperative KJLO mean values from each separate patient group without presenting the overall preoperative KJLO mean values: this also encumbers determining the measurement differences between the five KJLO measurement methods in this review.

To evaluate KJLO, the preferred, ideal measurement method is suggested not to be influenced by the single-leg or double-leg stance positions or by bipedal distance in the double-leg stance position on anteroposterior full-length standing radiographs. In that way, KJLO measurements can be compared between different patients using various radiographic techniques. Reproducibility of this preferred, ideal measurement method is likewise recommended, as it has good intraobserver and interobserver measurement reliability and is not influenced by confounding factors such as knee osteoarthritis severity and knee joint laxity grades.

Obtaining a 100 % anteroposterior projection full-length standing radiograph is crucial towards ensuring KJLO measurement accuracy in the coronal plane. To achieve this, it is recommended to use the position of a fully extended knee and the patella facing forward during the filming process.^39^^,^^49^^,^^50^ Besides standardizing the filming position, a lateral fluoroscopic control targeting the posterior femoral condyles helps guarantee a 100 % anteroposterior full-length standing radiograph.^51^

Based on the findings of this scoping review, more research is needed to determine the preferred, ideal KJLO measurement method that can be used regardless of the anteroposterior full-length standing radiographic technique used. A well-designed study that investigates preoperative KJLO measurement differences between the five KJLO measurement methods by the same radiographic technique would be required.

## 5 Conclusion

There is no consensus on how to measure KJLO or on which radiographic technique should be used. When measuring joint line orientation angles on anteroposterior full-length double-leg stance radiographs, controlling the bipedal distance with feet together is suggested when possible. Future research is needed to determine the measurement differences between the five KJLO measurement methods and to identify the preferred, ideal one.

## Consent to participate

Not applicable.

## Consent to publish

Not applicable.

## Author contributions

Conceptualization, RB, HV, IA and TX; methodology, HV, TX and IA; writing-original draft, TX; writing-review and editing, RB, IA and HV. All authors approved the final manuscript.

## Ethics approval

Not applicable.

## Data availability statement

Not applicable.

## Funding

The authors declare that no funds, grants, or other support were received during the preparation of this manuscript.

## Declaration of competing interest

The authors have no relevant financial or non-financial interests to disclose.
